# O034: Regional trends in enterobacteriaceae extended-spectrum beta-lactamase-producing (ESBLE) and methicillin-resistant staphylococcus aureus (MRSA) between 2007 and 2011

**DOI:** 10.1186/2047-2994-2-S1-O34

**Published:** 2013-06-20

**Authors:** I Arnaud, O Bajolet, X Bertrand, H Blanchard, E Caillat-Vallet, C Dumartin, M Eveillard, T Fosse, N Garreau, O Hoff, N Marty, S Maugat, E Reyreaud, A Savey, H Sénéchal, L Simon, E Sousa, D Trystram, B Coignard, V Jarlier, P Astagneau

**Affiliations:** 1 CClin-ARlin, France; 2 InVS, Paris, France

## Introduction

In France, for many years, MRSA infections have been decreasing whereas ESBLE (especially *E. coli*) infections have been increasing since 2006 inducing a specific national guideline for the prevention of ESBLE published in 2010.

## Objectives

The aim of this work is to illustrate the evolution of the regional incidences of MRSA and ESBLE between 2007 and 2011.

## Methods

A cohort of 454 Health care facilities (HCF) from 2007 to 2011 is issued from the national monitoring network of multidrug resistant bacteria in hospital (BMR-RAISIN) implemented since 2002. HCF participated within a 3 months survey period on a voluntary basis. Strains were isolated from sample issued for diagnostic purposes (a single strain of the same species per patient). Incidences of MRSA and ESBLE stratified by region were calculated per 1,000 patient-days (PD) from 2007 to 2011. An Univariate Poisson regression was used to estimate temporal trends.

## Results

From 2007 to 2011, 454 HCF participated each year in BMR-RAISIN network: 377 HCF with acute care unit (ACU), 165 with intensive care unit (ICU), and 354 with long-term care unit (R-LTCF). In almost all French regions (with more than 2 HCF participant), incidences of MRSA/1000 PD decreased significantly from 0.51 to 0.39 between 2007 and 2011 (p=10^-3^).

Overall incidences of MRSA decreased from 0.67 to 0.50 in ACU, from 1.75 to 1.04 in ICU and from 0.31 to 0.23 in R-LTCF. At the same time, EBLSE/1000 PD incidences increased significantly (p = 10^-3^) for all French regions from 0.24 to 0.50 globally, from 0.32 to 0.65 in ACU, 1.16 to 1.91 in ICU and from 0.14 to 0.28 in R-LTCF.

## Conclusion

Although the decrease of the MRSA incidences is encouraging, ESBLE incidences continue to increase strongly despite control efforts in all French regions between 2007 and 2011. This steady increase must incite implementation of HCF to upgrade their preventive recommended measures, in particular for contacts precautions and excreta management.

## Disclosure of interest

None declared.

